# Development and validation of a pre- and intra-operative scoring system that distinguishes between non-advanced and advanced axillary lymph node metastasis in breast cancer with positive sentinel lymph nodes: a retrospective study

**DOI:** 10.1186/s12957-022-02779-9

**Published:** 2022-09-28

**Authors:** Takeshi Murata, Chikashi Watase, Sho Shiino, Arisa Kurita, Ayumi Ogawa, Kenjiro Jimbo, Eriko Iwamoto, Masayuki Yoshida, Shin Takayama, Akihiko Suto

**Affiliations:** 1 Department of Breast Surgery, National Cancer Center Hospital, 5-1-1 Tsukiji, Chuo-ku, Tokyo, 104-0045 Japan; 2 Department of Diagnostic Pathology, National Cancer Center Hospital, 5-1-1 Tsukiji, Chuo-ku, Tokyo, 104-0045 Japan

**Keywords:** Breast cancer, Prediction of advanced lymph node metastasis, Scoring system, Sentinel lymph node metastasis, Preoperative

## Abstract

**Background:**

There are currently no scoring-type predictive models using only easily available pre- and intraoperative data developed for assessment of the risk of advanced axillary lymph node metastasis (ALNM) in patients with breast cancer with metastatic sentinel lymph nodes (SLNs). We aimed to develop and validate a scoring system using only pre- and intraoperative data to distinguish between non-advanced (≤ 3 lymph nodes) and advanced (> 3 lymph nodes) ALNM in patients with breast cancer with metastatic SLNs.

**Methods:**

We retrospectively identified 804 patients with breast cancer (cT1-3cN0) who had metastatic SLNs and had undergone axillary lymph node dissection (ALND). We evaluated the risk factors for advanced ALNM using logistic regression analysis and developed and validated a scoring system for the prediction of ALNM using training (*n* = 501) and validation (*n* = 303) cohorts, respectively. The predictive performance was assessed using the receiver operating characteristic (ROC) curve, area under the curve (AUC), and calibration plots.

**Results:**

Ultrasound findings of multiple suspicious lymph nodes, SLN macrometastasis, the ratio of metastatic SLNs to the total number of SLNs removed, and the number of metastatic SLNs were significant risk factors for advanced ALNM. Clinical tumor size and invasive lobular carcinoma were of borderline significance. The scoring system based on these six variables yielded high AUCs (0.90 [training] and 0.89 [validation]). The calibration plots of frequency compared to the predicted probability showed slopes of 1.00 (training) and 0.85 (validation), with goodness-of-fit for the model. When the cutoff score was set at 4, the negative predictive values (NPVs) of excluding patients with advanced ALNM were 96.8% (training) and 96.9% (validation). The AUC for predicting advanced ALNM using our scoring system was significantly higher than that predicted by a single independent predictor, such as the number of positive SLNs or the proportion of positive SLNs. Similarly, our scoring system also showed good discrimination and calibration ability when the analysis was restricted to patients with one or two SLN metastases.

**Conclusion:**

Our easy-to-use scoring system can exclude advanced ALNM with high NPVs. It may contribute to reducing the risk of undertreatment with adjuvant therapies in patients with metastatic SLNs, even if ALND is omitted.

**Supplementary Information:**

The online version contains supplementary material available at 10.1186/s12957-022-02779-9.

## Background

Recently, several trials have explored the safety of omitting axillary lymph node (ALN) dissection (ALND) and administering appropriate adjuvant therapy in patients with early breast cancer having limited axillary involvement [1–3]. However, precise information on the total number of involved lymph nodes cannot be obtained if ALND is omitted in patients with metastatic sentinel lymph nodes (SLNs). In previous studies, 13–33% of patients who had undergone ALND for SLN metastases had non-SLN metastases [1–3], and 13.7% of patients had ≥ 4 lymph node metastases [3]. When determining systemic therapy and regional irradiation strategies for patients with breast cancer, it is important to distinguish between non-advanced ALN status (i.e., 0–3 positive ALNs) and advanced ALN metastasis (ALNM) (i.e., ≥ 4 positive ALNs) [4]. Although performing ALND in patients with non-advanced ALNM may lead to overtreatment, omitting ALND in patients with advanced ALNM may lead to undertreatment. Therefore, it is important to accurately estimate whether a patient has non-advanced or advanced ALNM if omitting ALND in patients with metastatic SLNs. Several nomograms reportedly predict the risk of advanced ALNM in patients with metastatic SLNs; however, most nomograms use data obtained from surgery, such as pathological tumor size or lymphovascular invasion; therefore, it is difficult to use these nomograms intraoperatively [5–9]. Other nomograms use the total tumor load of SLNs evaluated by one-step nucleic acid amplification as a predictor [10, 11], but this method is not yet commonly used in clinical practice. Therefore, a model that can accurately predict the likelihood of a patient with metastatic SLN having advanced ALNM using only easily available preoperative and intraoperative data is required.

The number of suspicious nodes on axillary ultrasound (US) is related to the number of positive ALNs [12–14]. The histologic type of invasive lobular histology is related to advanced ALNM [7, 12, 15] as well. Although these factors may improve the predictive performance of ALN status, no model has been developed to predict advanced ALNM by combining preoperative data of axillary US imaging and histology with intraoperative data of SLNs.

We previously developed an easy-to-use scoring system with only preoperatively available data that could distinguish between advanced and non-advanced ALNM with a high degree of accuracy for patients with cT1-T3cN0-1 breast cancer [12]. However, some patients that were used to develop the previous predictive model had undergone ALND without SLN biopsy (SLNB) or had not undergone ALND when one to two SLNs were positive. Especially, we might have underestimated the total number of positive nodes because not all patients had undergone ALND. Additionally, the previous predictive model was not a model specific to clinically node-negative patients. Therefore, we conducted a new study to develop and evaluate a scoring model that can differentiate non-advanced and advanced ALNM cases with a combination of available preoperative and intraoperative data in clinically node-negative patients with metastatic SLNs who had undergone ALND.

## Methods

**Fig. 1:**
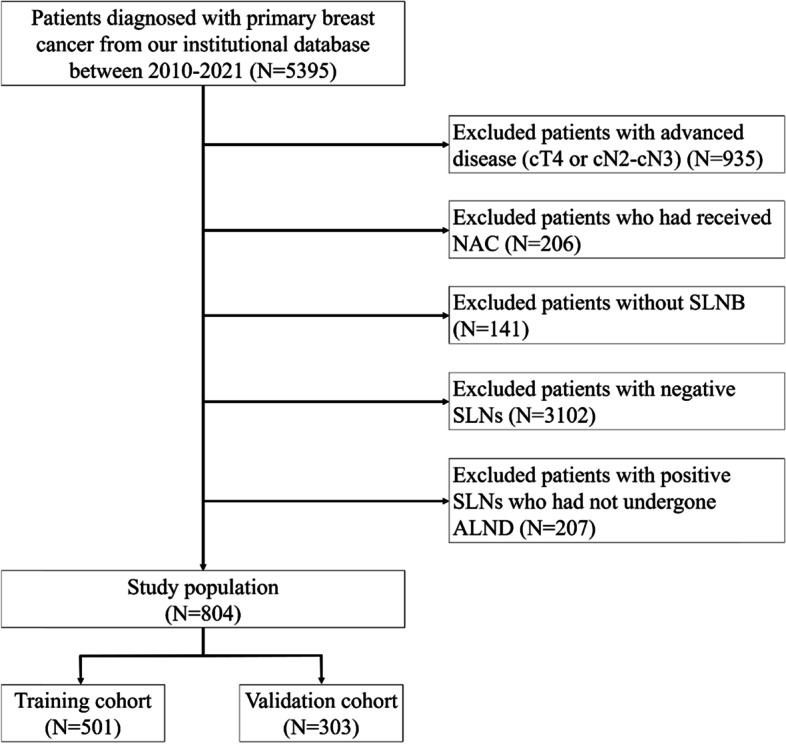
The flowchart of patients’ selection. NAC, neoadjuvant chemotherapy; SLNB, sentinel lymph node biopsy; SLN, sentinel lymph node; ALND, axillary lymph node dissection; AUC, area under the curve; CI, confidence interval

The dataset comprised consecutive patients who had operable primary breast cancer (clinical TNM stage, T1-T3, and N0 as per the 8th edition of the American Joint Committee of Cancer [AJCC] Cancer Staging Manual [16]) in our institutional database from January 2010 to October 2021. We excluded patients who had locally advanced disease (T4 or N2-3) and those who had received neoadjuvant chemotherapy (NAC). We also excluded patients who had not undergone SLNB and those with negative SLNs. Patients with positive SLNs who had not undergone ALND were also excluded. We identified 804 patients, who were randomly divided into the training and the validation cohorts in a ratio of 5:3 using the statistical software STATA SE version 13 (Stata Corp., College Station, TX, USA). The training and validation cohorts were used to develop the scoring system and for validation, respectively. The detailed exclusion criteria are presented in Fig. 1.

### Data collection

We obtained patients’ medical records regarding age at diagnosis, clinical tumor size evaluated by US, total number of suspicious lymph nodes detected by the axillary US, histologic tumor type, histologic tumor grade, estrogen receptor (ER) status, progesterone receptor (PR) status, human epidermal growth factor receptor 2 (HER2) status, Ki-67 level, and total number of metastatic lymph nodes in SLNB and ALND. ER and PR positivity were defined as immunohistochemical staining of > 1% of tumor cells. Hormone receptor (HR) positivity was defined as ER positivity and/or PR positivity. HER2 positivity was defined as a score of 3+ on immunohistochemistry or on amplification of fluorescence in situ hybridisation [16, 17]. Regarding the classification of Ki-67 levels, in the St. Gallen International Consensus Guidelines for treatment of early breast cancer 2021, the panel generally supported the recommendation that tumors with Ki-67 ≥ 30% receive chemotherapy, but the Ki-67 threshold for recommending chemotherapy in ER-positive cases could not be consistently defined as 10–25% [18]. Considering this point, we classified the tumors according to the Ki-67 level into the following three groups: ≤ 10%, 10–30%, and ≥ 30%. Diffuse cortical thickness > 5 mm, asymmetric cortical thickness > 3 mm, and complete or near-complete absence of fatty hilum on the axillary US were considered as suspicious lymph nodes [19, 20]. Fine-needle aspiration cytology was performed for suspicious ALNs. The most suspicious node was sampled in patients with multiple suspicious lymph nodes, and the number of suspicious nodes was recorded.

### Surgical procedure

SLNB was performed on all patients. SLNs were identified using lymphoscintigraphy (technetium-99m phyrate) and/or indocyanine green. Completion level I–II ALND was performed in all patients regardless of the metastatic size of the SLNs.

### Pathological evaluation

ALNs were evaluated using hematoxylin and eosin staining. Each ALN was classified as negative or positive for metastasis, and the number of metastatic ALNs was recorded. According to the current AJCC criteria [21], isolated tumor cells (deposit < 0.2 mm) were classified as negative (pN0i+), and micrometastatic deposits of 0.2–2.0 mm were classified as positive for metastasis (pN1mi). Pathologic nodal staging was determined by the number of metastatic ALNs as follows: pN0, none; pN1 (1–3 lymph node metastases), limited; and pN2-3 (> 3 lymph node metastases), advanced.

### Statistical analysis

Tumor characteristics and axillary US scans of patients in the training and validation cohorts were compared using the Mann–Whitney *U* test or chi-square test, as appropriate. In the training cohort, the odds ratios (ORs) and 95% confidence intervals (CIs) for advanced ALNM were calculated using univariate logistic regression analysis. Baseline variables (*P* < 0.10 on univariate analysis were assessed for multicollinearity using the variance inflation factor (VIF). A VIF of > 10 indicated multicollinearity between the variables [22]. Baseline variables (*P* < 0.10 on univariate analysis) were included in the multivariate regression analysis, which was used to devise the scoring system. For each of these variables (*P* < 0.10 on multivariate analysis), a score was calculated using the β-coefficient by the value rounded to the nearest integer as each score. The total score was derived from the sum of the scores for each variable. Then, discrimination and calibration accuracy of the scoring system was evaluated to distinguish between advanced and non-advanced ALNM cases. The discrimination ability of each scoring system was evaluated using the receiver operating characteristic (ROC) curve, and then assessed by calculating the area under the curve (AUC) with a 95% CI. Calibration was evaluated by comparing the expected number of patients with advanced ALNM (as predicted by each total score) with the observed number of patients with advanced ALNM. The calibration of our scoring system was tested by performing the Hosmer–Lemeshow goodness-of-fit test on the training cohort [23]. To evaluate the generalisability of the scoring system, a 5-fold cross-validation (CV) was performed [24] using the training cohort dataset.

The diagnostic performance of the scoring system for predicting advanced ALNM was estimated in terms of its AUC, sensitivity, specificity, positive predictive value (PPV), and negative predictive value (NPV) of each cutoff value of the scoring system. The feasibility of the scoring system was evaluated with the validation cohort.

All statistical analyses were conducted using the statistical software STATA SE version 13 (Stata Corp). *P <* 0.05 was set as the threshold for significance.

## Results

### Factors associated with advanced ALNM in the training cohort

Patient demographics and tumor characteristics of both the training and validation cohorts are summarized in Table 1. Patient and tumor characteristics were well-balanced between the training and validation cohorts.

**Table 1:** Baseline patient characteristics

Characteristics	Training Cohort (*n* = 501)		Validation cohort (*n* = 303)		*P* value
Age (years)					
Median (IQR)	52	(45–64)	52	(45–63)	0.971
Clinical tumor size, *n* (%)					0.086
≦1 cm	40	(8.0)	20	(6.6)	
1–2 cm	184	(36.7)	109	(36.0)	
2–3 cm	177	(35.3)	106	(35.0)	
3–4 cm	48	(9.6)	33	(10.9)	
4–5 cm	37	(7.4)	14	(4.6)	
> 5 cm	15	(3.0)	21	(6.9)	
Histologic grade, *n* (%)					0.577
1	107	(21.4)	66	(21.8)	
2	254	(50.7)	143	(47.2)	
3	140	(27.9)	94	(31.0)	
Histologic type, *n* (%)					0.615
IC-NST	420	(83.8)	254	(83.8)	
ILC	53	(10.6)	30	(9.9)	
IMPC	15	(3.0)	6	(2.0)	
Others	13	(2.6)	13	(4.3)	
Ki-67, *n* (%)					0.721
≤ 10%	80	(16.0)	46	(15.2)	
10–30%	214	(42.7)	121	(39.9)	
≥ 30%	145	(28.9)	94	(31.0)	
Unknown	62	(12.4)	42	(13.9)	
Subtype, *n* (%)					0.465
HR+/HER2−	407	(81.2)	237	(78.2)	
HR+/HER2+	45	(9.0)	32	(10.6)	
HR−/HER2+	14	(2.8)	14	(4.6)	
HR−/HER2-	35	(7.0)	20	(6.6)	
Breast surgery, *n* (%)					0.794
Total mastectomy	299	(59.7)	178	(58.8)	
Breast-conserving surgery	202	(40.3)	125	(41.3)	
No. of suspicious ALNs on US imaging^a^, *n* (%)					0.213
0	375	(74.9)	222	(73.3)	
1 (solitary)	86	(17.2)	46	(15.2)	
≥ 2 (multiple)	40	(8.0)	35	(11.6)	
Size of SLN metastasis, *n* (%)					0.301
ITC	86	(17.2)	41	(13.5)	
Micrometastasis	97	(19.4)	55	(18.2)	
Macrometastasis	318	(63.5)	207	(68.3)	
Ratio of no. of positive SLNs to total no. of SLNs, *n* (%)					0.254
< 0.5	244	(48.7)	135	(44.6)	
≥ 0.5	257	(51.3)	168	(55.5)	
Total no. of dissected SLNs					
Median (IQR)	3	(2–4)	3	(2–4)	0.908
No. of metastatic SLNs					0.380
0 (ITC)	86	(17.2)	41	(13.5)	
1	297	(59.3)	180	(59.4)	
2	89	(17.8)	58	(19.1)	
3	29	(5.8)	24	(7.9)	
Total no. of dissected ALNs					
Median (IQR)	17	(13–21)	17	(13–20)	0.967
Nodal status, *n* (%)					0.513
pN0i+	78	(15.6)	37	(12.4)	
pN1	348	(69.5)	217	(71.2)	
pN2	62	(12.4)	38	(13.0)	
pN3	13	(2.6)	11	(3.3)	

*IQR* interquartile range, *IC* invasive carcinoma, *NST* no special type, *ILC* invasive lobular carcinoma, *IMPC* invasive micropapillary carcinoma, *HR* hormone receptor, *HER2* human epidermal growth factor receptor 2, *ALN* axillary lymph node, *US* ultrasound, *ITC* isolated tumor cells, *SLN* sentinel lymph node

^a^Axillary lymph nodes were considered suspicious if at least one of the following were noted: diffuse cortical thickness > 5 mm, focal cortical thickness > 3 mm, and effacement or replacement of the fatty hilum on US imaging

The factors associated with advanced ALNM are summarized in Table 2. Univariate analysis revealed that the significant factors associated with advanced ALNM were the clinical tumor size, histologic type, type of surgery, axillary US findings of suspicious lymph nodes, size of SLN metastasis, ratio of the number of positive SLNs to the total number of SLNs removed, and number of positive SLNs. Variables with *P* < 0.10 in univariate analysis were assessed for multicollinearity (Table S1). No variable with a VIF > 10 was found, indicating no collinearity between the variables. In the multivariate analysis, patients with advanced ALNM were more likely to have US findings of multiple suspicious lymph nodes, SLN macrometastasis, higher ratio of the number of metastatic SLNs to the total number of SLNs removed, and two or three metastatic SLNs. A clinical tumor size of 4–5 cm or > 5 cm and a histologic type of invasive lobular carcinoma were of borderline significance.

**Table 2:** Results of univariate and multivariate analyses of factors associated with advanced ALNM (pN2-N3) in the training cohort

Characteristics		Univariate			Multivariate		
		OR	95% CI	***P*** value	OR	95% CI	***P*** value
Age, years							
< 40		1					
≥ 40		1.74	0.60–5.02	0.307			
Clinical tumor size							
≤ 1 cm		1			1		
1–2 cm		2.32	0.52–10.3	0.271	1.40	0.26–7.50	0.696
2–3 cm		3.59	0.82–15.8	0.090	1.20	0.22–6.44	0.828
3–4 cm		2.16	0.40–11.8	0.374	0.76	0.11–5.31	0.781
4–5 cm		11.6	2.41–55.6	0.002	4.94	0.80–30.5	0.086
> 5 cm		12.7	2.18–73.4	0.005	7.03	0.82–60.3	0.075
Histologic grade							
1		1					
2		1.37	0.70–2.67	0.362			
3		1.37	0.66–2.87	0.398			
Histologic type							
IC-NST		1			1		
ILC		2.88	1.50–5.52	0.001	2.31	0.94–5.69	0.068
IMPC		1.66	0.45–6.08	0.442	1.84	0.32–10.5	0.492
Others		0.60	0.08–4.78	0.634	0.38	0.04–3.83	0.441
Ki-67							
≤ 10%		1					
10–30%		1.24	0.60–2.57	0.568			
≥ 30%		1.10	0.50–2.43	0.805			
Subtype							
HR-/HER2−		1					
HR+/HER2−		0.80	0.32–2.02	0.640			
HR+/HER2+		1.01	0.31–3.24	0.988			
HR−/HER2+		0.39	0.04–3.59	0.405			
Breast surgery							
Breast-conserving surgery		1			1		
Total mastectomy		2.20	1.26–3.83	0.005	1.30	0.63–2.70	0.481
No. of suspicious ALNs on US imaging^a^							
0		1			1		
1 (solitary)		1.66	0.87–3.15	0.122	1.04	0.47–2.33	0.915
≥ 2 (multiple)		5.72	2.83–11.6	< 0.001	5.02	1.99–12.6	0.001
Size of SLN metastasis							
ITC, micrometastasis		1			1		
Macrometastasis		55.2	7.60–400	< 0.001	21.5	2.67–173	< 0.001
Ratio of no. of positive SLNs to total no. of SLNs							
< 0.5		1			1		
≥ 0.5		10.4	4.88–22.2	< 0.001	2.86	1.15–7.14	0.024
No. of positive SLNs							
0 (ITC only), 1		1			1		
2		8.25	4.50–15.1	< 0.001	3.05	1.47–6.34	0.003
3		39.7	15.9–98.9	< 0.001	15.7	5.56–44.2	< 0.001

*IC* invasive carcinoma, *NST* no special type, *ILC* invasive lobular carcinoma, *IMPC* invasive micropapillary carcinoma, *HR* hormone receptor, *HER2* human epidermal growth factor receptor 2, *ALND* axillary lymph node dissection, *ALN* axillary lymph node, *ALNM* axillary lymph node metastasis, *US* ultrasound, *ITC* isolated tumor cells, *SLN* sentinel lymph node

^a^Axillary lymph nodes were considered suspicious if at least one of the following were noted: diffuse cortical thickness > 5 mm, focal cortical thickness > 3 mm, and effacement or replacement of the fatty hilum on US imaging

### Scoring system for distinguishing between non-advanced and advanced ALNM

Using the results of the multivariate analysis, a scoring system was devised to assess the likelihood of advanced ALNM (Table 3). The factors included were the clinical tumor size, axillary US findings, histologic type, size of SLN metastasis, ratio of the number of positive SLNs to the total number of SLNs removed, and number of positive SLNs. A score was calculated using the β-coefficient from the multivariate analysis, and the value rounded to the nearest integer was used as each score. The total score was derived from the sum of the scores for each variable. The percentage of patients with advanced ALNM increased significantly as the total score increased (Table 4).

**Table 3:** Scoring system based on multivariate analysis in the training cohort

	Score	β-coefficient	Odds ratio	*P* value
Clinical tumor size (mm)				
≥ 1 cm	0	–	1	
1–2 cm	0	0.33	1.40	0.696
2–3 cm	0	0.19	1.20	0.828
3–4 cm	0	− 0.28	0.76	0.781
4–5 cm	2	1.60	4.94	0.086
> 5 cm	2	1.95	7.03	0.075
Histologic type				
IDC-NST	0	–	1	
Others	0	− 0.97	0.38	0.411
IMPC	0	0.84	1.84	0.492
ILC	1	0.61	2.31	0.068
No. of suspicious ALNs on US imaging^a^				
0	0	–	1	
1 (solitary)	0	0.04	1.04	0.915
≥ 2 (multiple)	2	1.61	5.02	0.001
Size of SLN metastasis				
ITC, micrometastasis	0	–	1	
Macrometastasis	3	3.07	21.5	< 0.001
Ratio of no. of positive SLNs to total no. of SLNs				
< 0.5	0	–	1	
≥ 0.5	1	1.05	2.86	0.024
No. of positive SLNs				
0 (ITC only), 1	0	–	1	
2	1	1.12	3.05	0.003
3	3	2.75	15.7	< 0.001

*IC* invasive carcinoma, *NST* no special type, *IDC* infiltrating ductal carcinoma, *ILC* invasive lobular carcinoma, *IMPC* invasive micropapillary carcinoma, *ALN* axillary lymph node, *US* ultrasound, *ITC* isolated tumor cells, *SLN* sentinel lymph node

^a^Axillary lymph nodes were considered suspicious if at least one of the following were noted: diffuse cortical thickness > 5 mm, focal cortical thickness > 3 mm, and effacement or replacement of the fatty hilum on US imaging

**Table 4:** Distribution of advanced ALNM (pN2-N3) stratified by total score in the training and validation cohorts

Total score^a^	Training cohort			Validation cohort		
	Patients (***n***)	Advanced ALNM		Patients (***n***)	Advanced ALNM	
0	114	0	(0.0)	52	0	(0.0)
1	40	0	(0.0)	24	0	(0.0)
2	22	0	(0.0)	15	0	(0.0)
3	81	2	(2.5)	50	1	(2.0)
4	91	9	(9.9)	55	5	(9.1)
5	75	19	(25.3)	48	11	(22.9)
6	22	6	(27.3)	14	5	(35.7)
7	36	22	(61.1)	27	16	(59.3)
8	11	9	(81.8)	11	6	(54.6)
9	6	5	(83.3)	6	4	(66.7)
10	2	2	(100)	1	1	(100)
11	1	1	(100)	0	0	(0.0)
Total	501	75	(15.0)	303	49	(16.2)

Data are presented as *n* (%)

*ALNM* axillary lymph node metastasis

^a^The scoring system is summarized in Table 3

### Predictive accuracy of the scoring system in the training cohort

**Fig. 2:**
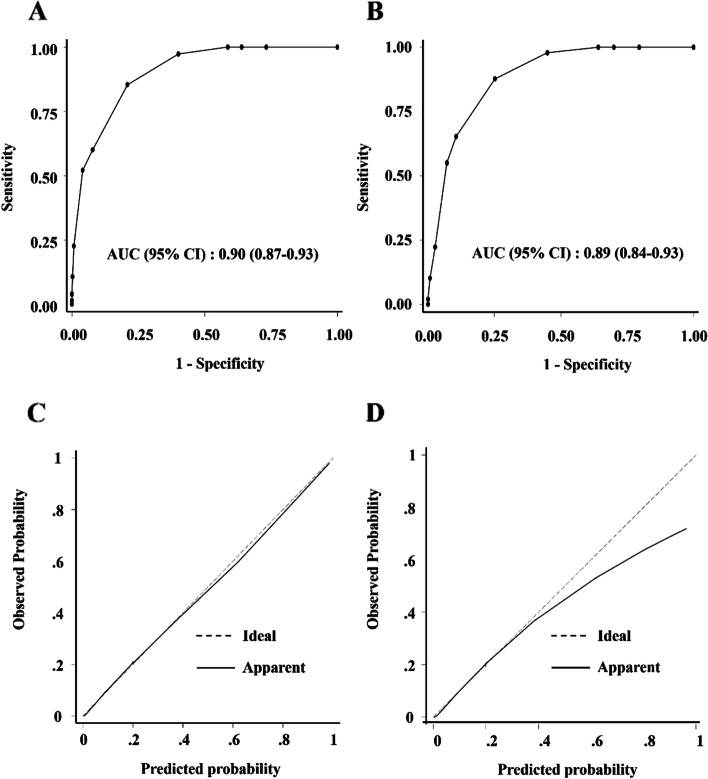
ROC curves of the scoring system for differentiating between non-advanced and advanced ALNM in the training cohort (**A**) and the validation cohort (**B**), and calibration plots of the system for the training cohort (**C**) and the validation cohort (**D**). The Hosmer–Lemeshow test indicated goodness-of-fit for the model in the training (*χ*^2^ = 3.09, *P* = 0.69) and validation cohorts (*χ*^2^ = 9.44, *P* = 0.31). The calibration plot of the observed frequency compared to the predicted probability of the scoring model showed slopes of 1.000 for the training cohort (**C**) and 0.852 for the validation cohort (**D**). ROC, receiver operating characteristic; ALNM, axillary lymph node metastasis; AUC, area under the curve; CI, confidence interval

The ROC curves for the scoring system in the training cohort are shown in Fig. 2A. The AUC was 0.90, and the 5-fold CV showed a mean AUC of 0.90. The calibration plots of frequency compared to the predicted probability of the scoring model showed a slope of 1.00 for the training cohort (Fig. 2C), and the Hosmer-Lemeshow test indicated goodness-of-fit for the model in the training cohort (*P* = 0.69). The predictive accuracy of the scoring system for differentiating between non-advanced and advanced ALNM cases at each cutoff value is presented in Table 5. The AUC value was highest when the cutoff value was a total score of 4. At this score, the NPV for excluding advanced ALNM was 96.8%, and the sensitivity, specificity, and PPV were 85.3%, 79.1%, and 41.8%, respectively.

**Table 5:** Predictive ability of the scoring system to differentiate between non-advanced and advanced ALNM (pN2-N3) at each cutoff point in the training and validation cohorts

Total score cutoff^a^	Sensitivity (%)	Specificity (%)	PPV (%)	NPV (%)	AUC (95% CI)	*P* value
Training Cohort						
≦ 2	100.0	41.3	23.1	100.0	0.71 (0.68–0.73)	< 0.0001
≦ 3	97.3	59.9	29.9	99.2	0.79 (0.76–0.82)	0.0869
≦ 4	85.3	79.1	41.8	96.8	0.82 (0.78–0.87)	Reference
≦ 5	60.0	92.3	57.7	92.9	0.76 (0.70–0.82)	0.0218
≦ 6	52.0	96.0	69.6	91.9	0.74 (0.68–0.80)	0.0044
≦ 7	22.7	99.3	85.0	87.9	0.61 (0.56–0.66)	< 0.0001
≦ 8	10.7	99.8	88.9	86.4	0.55 (0.52–0.59)	< 0.0001
Validation Cohort						
≦ 2	100.0	35.8	23.1	100.0	0.68 (0.65–0.71)	< 0.0001
≦ 3	98.0	55.1	29.6	99.3	0.76 (0.71–0.80)	0.0366
≦ 4	87.8	74.8	40.2	96.9	0.81 (0.76–0.87)	Reference
≦ 5	65.3	89.0	53.3	93.0	0.76 (0.69–0.83)	0.1171
≦ 6	55.1	92.5	58.7	91.4	0.74 (0.67–0.81)	0.00375
≦ 7	22.5	97.2	61.1	86.7	0.60 (0.54–0.66)	< 0.0001
≦ 8	10.2	99.2	71.4	85.1	0.55 (0.50–0.59)	< 0.0001

*ALNM* axillary lymph node metastasis, *AUC* area under the receiver operating characteristic curve, *CI* confidence interval, *NPV* negative predictive value, *PPV* positive predictive value

^a^The scoring system is summarized in Table 3﻿

### Performance of the scoring system for the validation cohort

The percentage of patients with advanced ALNM in the validation cohort also increased significantly as the total score increased (Table 4). The ROC curve for the scoring system in the validation cohort is shown in Fig. 2B. The AUC was 0.89, and the calibration plots of frequency compared to the predicted probability of the scoring model showed a slope of 0.852 (Fig. 2D). The Hosmer–Lemeshow test indicated goodness-of-fit for the model in the validation cohort (*P* = 0.31). At a total score of 4 points, the AUC was 0.81 (95% CI, 0.76–0.87); the NPV for excluding advanced ALNM was 96.9%; and sensitivity, specificity, and PPV were 87.8%, 74.8%, and 40.2%, respectively (Table 5).

## Discussion

In the present study, we developed a scoring system using available preoperative clinicopathological factors and the intraoperative SLN status to differentiate between non-advanced and advanced ALNM in patients with breast cancer having positive SLNs following ALND. We evaluated the predictive accuracy of the scoring system by assessing the discrimination and calibration ability in the training cohort and then evaluated its feasibility using the validation cohort. The results revealed that the scoring system had a high predictive performance. Therefore, our scoring system may identify patients with positive SLNs who are likely to have non-advanced ALNM.

The tumor size, histologic type, size of SLN metastasis, number of positive SLNs, proportion of positive SLNs, and suspicious lymph nodes on the axillary US are potential factors that would be useful in predicting the likelihood of advanced ALNM [5–9, 11, 12], which was confirmed by our study. Therefore, we confirmed the non-collinearity between these variables before developing the scoring system.

The pathological tumor size has often been reported as a factor for predicting advanced ALNM [5–8, 11], while the clinical tumor size has been less frequently reported [9, 12]. Although pathological tumor size is a more accurate factor than clinical tumor size, it is unavailable preoperatively; therefore, we used clinical tumor size evaluated by US to develop our scoring system, which assigned 2 and 0 points for a clinical tumor size > 4 cm and ≤ 4 cm, respectively. Among the 717 patients in our entire cohort whose clinical tumor size was estimated to be < 4 cm, only 71 (9.9%) had a pathological tumor size > 4 cm (data not shown). Thus, the NPV of patients whose clinical tumor size was estimated to be ≤ 4 cm was as high as 90.1%, suggesting that the risk of underestimating the tumor size was low. The clinical tumor size is easy to estimate by US, which is an advantage of our scoring system.

ILC was an independent risk factor for advanced ALNM in our study. US findings of axillary nodes coupled with FNA have been reported to be useful in predicting heavy axillary burden [25]. Advanced ALNM with false-negative US findings was more prevalent in patients with ILC than in those with infiltrating ductal carcinoma (IDC) because of the morphological features of ILC [26, 27]. Fine-needle aspiration biopsy of suspicious ALNs on US was less sensitive in patients with ILC than in those with IDC [28, 29]. Therefore, we believe that the inclusion of histologic type as a predictive factor in the scoring system is an important complement to US findings.

A suspicious ALN on US often corresponds to an SLN. Considering that the false-negative rate for SLNB is 7.3–9.8% [30, 31], adding axillary US findings to the scoring system may reduce the risk of underestimating non-SLN metastasis. The AUC value for predicting advanced ALNM using our scoring system was significantly higher than that predicted by a single independent predictor, such as the number of positive SLNs or the proportion of positive SLNs (Figure S1).

In the analysis restricted to patients with one or two SLN metastases, our scoring system showed similarly good discrimination and calibration abilities (Figure S2). Specifically, 386 (77.0%) of 501 patients in the training cohort and 238 (78.5%) of 303 patients in the validation cohort had 1–2 SLN metastases, while 54 (14.0%) of 386 patients in the training cohort and 31 (13.0%) of 238 patients in the validation cohort had advanced ALNM; these frequencies were similar to those previously reported [3]. Assuming that these patients with advanced ALNM undergo only SLNB, the indications for postoperative chemotherapy and regional irradiation may be determined based on the underestimated lymph node status of the SLNB, thereby resulting in undertreatment.

In the training cohort, when the cutoff point of our scoring system was set to 4, 262 (67.9%) patients with 1–2 metastatic SLNs had a score of ≤ 4 points, and the NPV was 95.8% (Tables S2 and S3). A high NPV indicated that our scoring system accurately detected patients with 1–2 metastatic SLNs but a low risk of advanced ALNM. Therefore, even if ALND is omitted in patients with a score ≤ 4 and the indication for postoperative chemotherapy or regional irradiation is determined based only on SLNB results, the risk of undertreatment is low. Although no adverse prognostic effect of ALND omission has been observed [3], at each individual patient level, underestimating the number of lymph node metastases due to ALND omission may affect decisions regarding adjuvant treatment, leading to a risk of undertreatment. As our study was not a prospective study, the effectiveness of using the total score to determine whether ALND should be performed (and whether adjuvant therapy should be administered) is unclear. However, the ability of our scoring system to intraoperatively predict the risk of non-advanced and advanced ALNM at the individual level in patients with 1–2 metastatic SLNs may help reduce the risk of undertreatment with adjuvant therapy even if ALND is omitted.

The strength of our model is that it is based on available clinicopathological features from preoperative and intraoperative evaluations conducted in routine clinical oncology practice. The first advantage of including US findings in the predictive model is that US is a non-invasive, reproducible, and low-cost diagnostic tool. Second, while axillary assessment by magnetic resonance imaging is affected by body mass index and has decreased sensitivity in obese patients [32], US has been reported to have similar sensitivity in obese and non-obese patients and better specificity in obese patients [33]. All patients underwent level I–II ALND completion, which provided accurate information regarding ALNM. Additionally, the scoring system-type prediction model is relatively simple compared to the nomogram-type model and is easily implemented in daily clinical practice.

This study had several limitations. First, it was performed at a single institution. Second, although we collected data from consecutive patients with invasive breast carcinoma, we did not control for selection bias. Patients who had undergone NAC were excluded. Patients with HR−/HER2+ and HR−/HER2− tended to receive NAC compared to those with HR+/HER2−, even if they were clinically node-negative, and the inclusion of these patients might have affected our results.

## Conclusions

We developed a scoring system to accurately differentiate non-advanced ALNM from advanced ALNM in patients with breast cancer demonstrating SLN metastasis. This scoring system is easy to use, requires only preoperative and intraoperative available data, and can exclude advanced ALNM with a high NPV. Hence, it may contribute to reducing the risk of undertreatment with adjuvant therapy in patients with metastatic SLNs, even if ALND is omitted.

## Supplementary Information

**Additional file 1: Figure S1.** Comparison of ROC curves of the scoring system between the training (A) and validation (B) cohorts and the analysis of independent factors to differentiate between non-advanced and advanced ALNM in both these cohorts. Figure S2. ROC curves of the scoring system compared between the training (A) and validation cohorts (B) and presentation of independent factors for differentiating between non-advanced and advanced ALNM in the two cohorts and calibration plots of the scoring system for the training cohort (C) and the validation cohort (D) in patients with one or two metastatic SLNs.

**Additional file 2: Table S1.** Evaluation of multivariable collinearity with *P* < 0.1 in the univariate analysis. **Table S2.** Distribution of advanced ALNM (pN2-N3) stratified by the total score in the training and validation cohorts in patients with one or two metastatic SLNs. **Table S3.** Predictive ability of the scoring system to differentiate between non-advanced and advanced ALNM at each cutoff point in the training cohort (A) and validation cohort (B) in patients with one or two metastatic sentinel lymph nodes.

## Data Availability

The datasets analyzed during the current study are available from the corresponding author on reasonable request.

## Abbreviations

AJCC: American Joint Committee of Cancer
ALN: Axillary lymph node
ALND: Axillary lymph node dissection
ALNM: Axillary lymph node metastasis
AUC: Area under the receiver operating characteristic curve
CI: Confidence interval
CV: Cross-validation
ER: Estrogen receptor
HER2: Human epidermal growth factor receptor 2
HR: Hormone receptor
IDC: Invasive ductal carcinoma
ILC: Invasive lobular carcinoma
LVI: Lymphovascular invasion
NAC: Neoadjuvant chemotherapy
NPV: Negative predictive value
OR: Odds ratio
PPV: Positive predictive value
PR: Progesterone receptor
ROC: Receiver operating characteristic
SLN: Sentinel lymph node
SLNB: Sentinel lymph node biopsy
US: Ultrasound
VIF: Variance inflation factor
