# Supplementary material for: Development and validation of a pre- and intra-operative scoring system that distinguishes between non-advanced and advanced axillary lymph node metastasis in breast cancer with positive sentinel lymph nodes: a retrospective study
Source: World J Surg Oncol. 2022 Sep 28;20:314. doi: 10.1186/s12957-022-02779-9 (PMC9516796; doi:10.1186/s12957-022-02779-9)
Supplement: Supplementary file 1 — Additional file 1: Figure S1. Comparison of ROC curves of the scoring system between the training (A) and validation (B) cohorts and the analysis of independent factors to differentiate between non-advanced and advanced ALNM in both these cohorts. Figure S2. ROC curves of the scoring system compared between the training (A) and validation cohorts (B) and presentation of independent factors for differentiating between non-advanced and advanced ALNM in the two cohorts and calibration plots of the scoring system for the training cohort (C) and the validation cohort (D) in patients with one or two metastatic SLNs. [file 12957_2022_2779_MOESM1_ESM.docx]

**Fig. S1: Comparison of ROC curves of the scoring system between the training (A) and validation (B) cohorts and the analysis of independent factors to differentiate between non-advanced and advanced ALNM in both these cohorts**


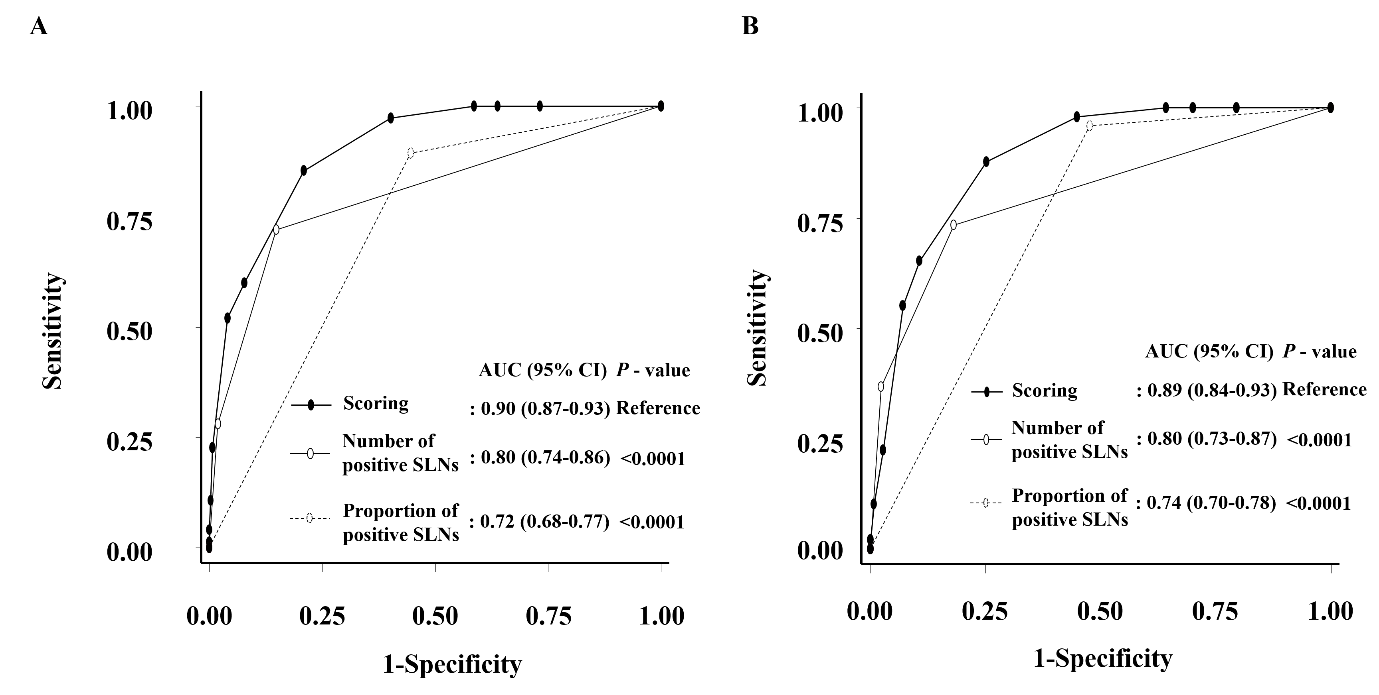


In the training cohort (A), the AUC of the scoring system, number of positive SLNs, and proportion of positive SLNs were 0.90 (95% CI, 0.87–0.93), 0.80 (95% CI, 0.74–0.86), and 0.72 (0.68–0.77), respectively. In the validation cohort (B), the AUC of the scoring system, number of positive SLNs, and proportion of positive SLNs were 0.89 (95% CI, 0.84–0.93), 0.80 (95% CI, 0.73–0.87), and 0.74 (0.70–0.78), respectively.

ROC, receiver operating characteristic; ALNM, axillary lymph node metastasis; AUC, area under the receiver operating characteristic curve; CI, confidence interval; SLN, sentinel lymph node.

**Fig. S2: ROC curves of the scoring system compared between the training (A) and validation cohorts (B)and presentation of independent factors for differentiating between non-advanced and advanced ALNM in the two cohorts and calibration plots of the scoring system for the training cohort (C) and the validation cohort (D) in patients with one or two metastatic SLNs**


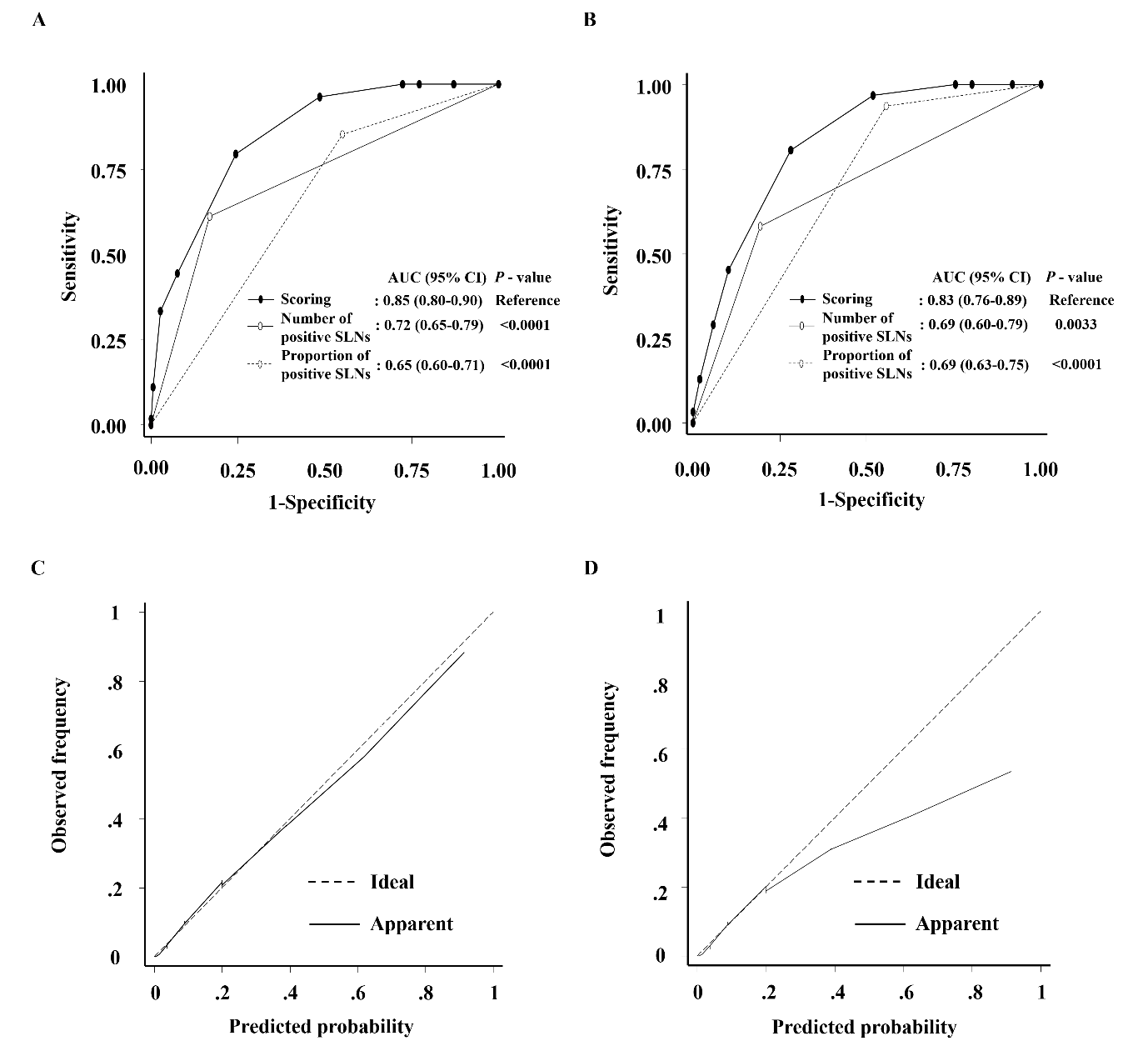


In the training cohort (A), the AUC of the scoring system, number of positive SLNs, and proportion of positive SLNs were 0.85 (95% CI, 0.80–0.90), 0.72 (95% CI, 0.65–0.79), and 0.65 (0.60–0.71), respectively. In the validation cohort (B), the AUC of the scoring system, number of positive SLNs, and proportion of positive SLNs were 0.83 (95% CI, 0.76–0.89), 0.69 (95% CI, 0.60–0.79), and 0.69 (0.63–0.75), respectively.

The Hosmer–Lemeshow test indicated goodness-of-fit for the scoring model in the training (χ^2^ = 3.56, *P* = 0.61) and validation cohorts (χ^2^ = 8.02, *P* = 0.33). The calibration plots of observed frequencies versus predicted probabilities of the scoring model showed slopes of 0.984 for the training cohort (C) and 0.769 for the validation cohort (D).

ROC, receiver operating characteristic; ALNM, axillary lymph node metastasis; AUC, area under the receiver operating characteristic curve; CI, confidence interval; SLN, sentinel lymph node.
