# Supplementary material for: Development and validation of a pre- and intra-operative scoring system that distinguishes between non-advanced and advanced axillary lymph node metastasis in breast cancer with positive sentinel lymph nodes: a retrospective study
Source: World J Surg Oncol. 2022 Sep 28;20:314. doi: 10.1186/s12957-022-02779-9 (PMC9516796; doi:10.1186/s12957-022-02779-9)
Supplement: Supplementary file 2 — Additional file 2: Table S1. Evaluation of multivariable collinearity with P < 0.1 in the univariate analysis. Table S2. Distribution of advanced ALNM (pN2-N3) stratified by the total score in the training and validation cohorts in patients with one or two metastatic SLNs. Table S3. Predictive ability of the scoring system to differentiate between non-advanced and advanced ALNM at each cutoff point in the training cohort (A) and validation cohort (B) in patients with one or two metastatic sentinel lymph nodes. [file 12957_2022_2779_MOESM2_ESM.docx]

**Table S1.** **Evaluation of multivariable collinearity with *P* < 0.1 in the univariate analysis**

| Variables | VIF |
| --- | --- |
| Clinical tumour size | 1.19 |
| Histologic type | 1.01 |
| No. of suspicious ALNs on US imaging | 1.09 |
| Size of SLN metastasis | 1.34 |
| No. of positive SLNs | 1.43 |
| Ratio of no. of positive SLNs to total no. of SLNs | 1.31 |
| Breast surgery | 1.15 |

VIF, variance inflation factor; ALN, axillary lymph node; US, ultrasound; SLN, sentinel lymph node.

**Table S2.** **Distribution of advanced ALNM (pN2-N3) stratified by the total score in the training and validation cohorts in patients with one or two metastatic SLNs**

|  | Training Cohort | | | Validation Cohort | | |
| --- | --- | --- | --- | --- | --- | --- |
| Total Score* | Patients (n) | Advanced ALNM, n (%) | | Patients (n) | Advanced ALNM, n (%) | |
| 0 | 43 | 0 | (0.0) | 17 | 0 | (0.0) |
| 1 | 33 | 0 | (0.0) | 24 | 0 | (0.0) |
| 2 | 16 | 0 | (0.0) | 10 | 0 | (0.0) |
| 3 | 81 | 2 | (2.5) | 50 | 1 | (2.0) |
| 4 | 89 | 9 | (10.1) | 54 | 5 | (9.3) |
| 5 | 75 | 19 | (25.3) | 48 | 11 | (22.9) |
| 6 | 22 | 6 | (27.3) | 14 | 5 | (35.7) |
| 7 | 19 | 12 | (63.2) | 13 | 5 | (38.5) |
| 8 | 7 | 5 | (71.4) | 7 | 3 | (42.9) |
| 9 | 1 | 1 | (100) | 1 | 1 | (100) |
| Total | 386 | 54 | (14.0) | 238 | 31 | (13.0) |

ALNM, axillary lymph node dissection; SLN, sentinel lymph node

*The scoring system is summarised in Table 3.

**Table S3.** **Predictive ability of the scoring system to differentiate between non-advanced and advanced ALNM at each cutoff point in the training cohort (A) and validation cohort (B) in patients with one or two metastatic sentinel lymph nodes**

**(A)**

| Total Score Cutoff* | Sensitivity (%) | Specificity (%) | PPV (%) | NPV (%) | AUC (95% CI) | *P*-value |
| --- | --- | --- | --- | --- | --- | --- |
| ≦ 2 | 100.0 | 27.7 | 18.3 | 100.0 | 0.64 (0.61–0.66) | <0.0001 |
| ≦ 3 | 96.3 | 51.5 | 24.4 | 98.8 | 0.74 (0.70–0.78) | 0.1872 |
| ≦ 4 | 79.6 | 75.6 | 34.7 | 95.8 | 0.78 (0.72–0.84) | Ref |
| ≦ 5 | 44.4 | 92.5 | 49.0 | 92.5 | 0.68 (0.62–0.75) | 0.0077 |
| ≦ 6 | 33.3 | 97.3 | 66.7 | 90.0 | 0.65 (0.59–0.72) | 0.0006 |
| ≦ 7 | 11.1 | 99.4 | 75.0 | 87.3 | 0.55 (0.51–0.60) | <0.0001 |
| ≦ 8 | 1.9 | 100.0 | 100.0 | 86.2 | 0.51 (0.49–0.53) | <0.0001 |

**(B)**

| Total Score Cutoff* | Sensitivity (%) | Specificity (%) | PPV (%) | NPV (%) | AUC (95% CI) | *P*-value |
| --- | --- | --- | --- | --- | --- | --- |
| ≦ 2 | 100.0 | 24.6 | 16.6 | 100.0 | 0.62 (0.59–0.65) | 0.0005 |
| ≦ 3 | 93.6 | 48.3 | 21.3 | 98.0 | 0.71 (0.65–0.76) | 0.1907 |
| ≦ 4 | 80.7 | 72.0 | 30.1 | 96.1 | 0.76 (0.69–0.84) | Ref |
| ≦ 5 | 41.9 | 89.4 | 37.1 | 91.1 | 0.66 (0.57–0.75) | 0.0216 |
| ≦ 6 | 29.0 | 93.7 | 40.9 | 89.8 | 0.61 (0.53–0.70) | 0.0018 |
| ≦ 7 | 12.9 | 98.1 | 50.0 | 88.3 | 0.55 (0.49–0.62) | <0.0001 |
| ≦ 8 | 3.2 | 100.0 | 100.0 | 87.3 | 0.52 (0.48–0.55) | <0.0001 |

ALN, axillary lymph node; AUC, area under the receiver operating characteristic curve; CI, confidence interval; NPV, negative predictive value; PPV, positive predictive value; SLN, sentinel lymph node.

*The scoring system is summarised in Table 3.
